# Fibrin glue in ophthalmology

**DOI:** 10.4103/0301-4738.55079

**Published:** 2009

**Authors:** Anita Panda, Sandeep Kumar, Abhiyan Kumar, Raseena Bansal, Shibal Bhartiya

**Affiliations:** Dr. Rajendra Prasad Centre for Ophthalmic Sciences, All India Institute of Medical Sciences, Ansari Nagar, New Delhi, India; 1 Department of Ophthalmology, Subharti Institute of Medical Sciences, Meerut, Uttar Pradesh, India; 2 Dr. Patnaik's Laser Eye Institute, New Delhi, India

**Keywords:** Blepharoplasty, cyanoacrylate glue, fibrin glue, fibrinogen, pterygium, stem cells, thrombin

## Abstract

Suturing is a time consuming task in ophthalmology and suture induced irritation and redness are frequent problems. Postoperative wound infection and corneal graft rejection are examples of possible suture related complications. To prevent these complications, ophthalmic surgeons are switching to sutureless surgery. A number of recent developments have established tissue adhesives like cyanoacrylate glue and fibrin glue as attractive alternatives to sutures. A possible and promising new application for tissue adhesives is to provide a platform for tissue engineering. Currently, tissue glue is being used for conjunctival closure following pterygium and strabismus surgery, forniceal reconstruction surgery, amniotic membrane transplantation, lamellar corneal grafting, closure of corneal perforations and descematoceles, management of conjunctival wound leaks after trabeculectomy, lid surgery, adnexal surgery and as a hemostat to minimise bleeding. The purpose of this review is to discuss the currently available information on fibrin glue.

Suturing is a time consuming process for which surgeons are in search of an ideal alternative. An ideal suture is one which is easy to handle, non-allergenic, affordable and does not promote infection. Besides, none of the sutures currently available fulfill the requirements of an ideal suture. To overcome these shortcomings, tissue adhesives are being increasingly used. An ideal tissue adhesive should have the following properties.[1]

- Must allow sufficient working time before inducing firm adhesion.
- Must have adequate tensile strength to maintain wound integrity.
- Must be biocompatible
- Should be clear enough to permit vision.
- Should be permeable to fluids and metabolites to prevent necrosis.
- Must not induce inflammation.
- Must disappear eventually to permit healing at the interface.
- Should not carry the risk of transferring an infectious agent.
- Accessible and affordable

The aim of the present review is to discuss the currently used tissue adhesive at length.

## Types

The two basic categories of tissue adhesives are - synthetic (commonest is n-butyl-2-cyanoacrylate) and biological (fibrin glue).[1]

In addition to these two tissue adhesives, newer adhesives available for surgeons are:

- Gelatin and thrombin products
- Albumin and glutaraldehyde products
- Polyethylene glycol polymers

Each of these products is unique in terms of advantages and limitations and consequently used for different indications. Of all tissue adhesives, more reports available in world literature are about cyanoacrylate and fibrin glue. In this review, we shall be discussing about fibrin glue.

## Cyanoacrylate

Cyanoacrylate-based glues have traditionally been the most widely used glues for ophthalmic surgery.[2] Cyanoacrylates require minimal hydration to polymerise and set. They can only be used externally because they induce inflammation. The tensile strength of the bonding is one of the highest of all glues. The major draw back of cyanoacrylate glue is that they form a solid, impermeable mass *in situ*. This persists as a foreign body causing inflammatory reactions like giant papillary conjunctivitis[3] and corneal neovascularization. They are also impermeable to fluids and metabolites. Though these disadvantages preclude its intraocular use, they are not very significant if the glue is applied superficially.[4]

## Fibrin Sealants

Fibrin glue is a blood-derived product that is absorbable, relatively easy to use, and can be kept at room temperature or in a refrigerator. Although the use of fibrin as a biologic adhesive was first introduced in 1909, it was not until 1944 that Tidrick *et al*. used fibrin for skin graft fixation.[5] Also it was in early forties that fibrin glue was introduced to ophthalmology to fixate penetrating corneal grafts in rabbits.[6]

Fibrin glue is a biological tissue adhesive which imitates the final stages of the coagulation cascade when a solution of human fibrinogen is activated by thrombin (the two components of fibrin glue).[7–9] Fibrin glue includes a fibrinogen component and a thrombin component, both prepared by processing plasma. It can be prepared at a blood transfusion center[10–12] or from patients own blood[13–26] or obtained as a commercially available preparation.[27] When it is derived from individual volunteer donations, it may have a low concentration of fibrinogen.[28–30] The commercially available products are produced from pools of plasma, usually contain high yields of fibrinogen and, consequently, produce firm coagulums. Unlike cyanoacrylate glue, fibrin glue forms a smooth seal along the entire length of the wound edge and thereby provides greater postoperative comfort to the patient with fewer complications.[31]

## Mechanisms of action

When human tissue is injured, bleeding ensues and then ceases due to formation of a blood clot. This is the initial mechanism of natural wound closure. Clot is formed as a product of the final common pathway of blood coagulation. Fibrin glue mimics this coagulation cascade resulting in its adhesive capability.[7]

**Figure 1:**
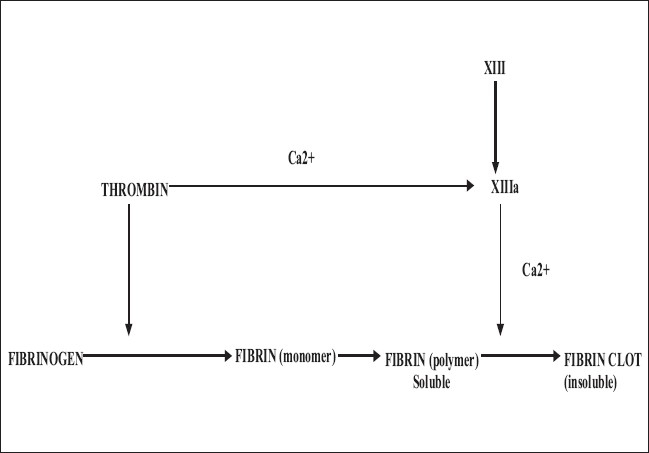
Final common pathway of coagulation cascade

Once the coagulation cascade is triggered, activated factor X selectively hydrolyses prothrombin to thrombin. In the presence of thrombin, fibrinogen is converted to fibrin. Thrombin also activates factor XIII (present in the fibrinogen component of the glue), which stabilizes the clot, by promoting polymerization and cross linking of the fibrin chains to form long fibrin strands in the presence of calcium ions [Fig. 1]. This is the final common pathway for both the extrinsic and intrinsic pathways of coagulation *in vivo*, which is mimicked by fibrin glue to induce tissue adhesion.

There is subsequent proliferation of fibroblasts and formation of granulation tissue within hours of clot polymerization. Clot organization is complete two weeks after application. The resultant fibrin clot degrades physiologically.

## Methods of preparation

Numerous techniques have been used to prepare fibrin glue, either from homologous or autologous plasma.[32–40] The autologous source avoids any possible risk of viral transmission. Homologous fibrin glue is prepared from donors screened like other blood products, followed by inactivation of viruses by solvent / detergent treatment.[41,42]

The plasma is centrifuged to produce a precipitate containing fibrinogen and a supernatant containing the thrombin. The precipitate is resuspended in a small volume of the supernatant and used as the fibrinogen component. The supernatant is further treated by clotting to convert residual fibrinogen to fibrin followed by its filtration to isolate the fibrin. The resulting serum is used as the thrombin component.

The various methods of preparation are:

- Fibrinogen: Modified Hartman's Procedure[13]
- Thrombin: Armand J Quick method[32]
- Fibrinogen rich concentrate[25,43–48]
- Preparation during emergency need[18,44]

## Large-scale preparation of thrombin from human plasma

Recently Aizawa *et al*. have discussed the preparation of thrombin for large scale use.[49] Simlarly, Alston *et al*. described another cost effective method of autologus fibrin sealant from protamine precipitated fibrinogen concentrate.[50] De Somer *et al*. have demonstrated the mechanical and chemical characterstics of autologus surgical glue made by mixing ultra filtered plasma with gluteraldehyde.[51]

## Commercially available fibrin glue

Tisseel VH Fibrin sealant (Baxter AG, Vienna, Austria) is a commercially available fibrin adhesive approved by the US Food and Drug Administration as an adjunct to hemostasis. The kit contains the following in separate vials.

1. Large Blue Bottle: Sealer protein concentrate (Human), Freeze dried, vapour treated, containing:
  - Clottable protein - 75 to 115mg
  - Fibrinogen - 70 to 110mg
  - Plasma fibronectin - 2 to 9 mg
  - Factor XIII - 10 to 50 IU
  - Plasminogen - 40 to 120 µg (microgram)
2. Small Blue Bottle: Aprotinin solution, bovine 3000 KIU /ml
3. White Bottle: Thrombin 4 (bovine), freeze dried reconstituted contains 4IU/ml
4. Large Black Bottle: Thrombin 500 (bovine), freeze dried reconstituted contains 500 IU/ml
5. Small Black Bottle: Calcium chloride solution, 40mmol/L
  1+2 (Fibrin component)
  3+5 (Thrombin component)- Used for slow release
  4+5 (Thrombin component)- Used for rapid release

It is advisable to maintain the cold chain, i.e., to keep the temperature well regulated constantly from the time after preparation till use. Before use, the syringes containing two components of fibrin glue, namely, Thrombin (Black) and Fibrinogen (Blue) are taken out from the deep freeze and thawed to room temperature.

The fibrin sealant (Tisseel VH, Baxter Healthcare Corp, Deerfield, IL) is prepared according to the manufacturer's directions. In brief, color-coded vials (color code-Black and Blue) are warmed for several minutes in a patented fibrotherm device. The procedure requires the addition of the fibrinolysis inhibitor, Aprotinin, to the sealer protein concentrate vial followed by warming. While this solution is being stirred, the second component is prepared by injecting the contents of calcium chloride vial into the Thrombin vial (Thrombin 500 or Thrombin 4, depending upon whether an early or a delayed clot is required) which is then warmed. Only a small amount of the thrombin-calcium chloride solution is required to drive the reaction to fibrin formation. To slow the process of fibrin formation, only 0.1ml of the thrombin-calcium chloride solution is withdrawn into a disposable syringe to which 0.9ml of balanced salt solution (Acorn Inc, Decatur, IL, USA) is added to achieve a 1:10 dilution. This syringe is placed into the duploject injector along with a parallel disposable syringe containing the fibrin sealer protein and fibrinolysis inhibitor. A mixer nosecone, topped by a blunt applicator needle, is attached to the 2-syringe nozzle to facilitate mixing of the two syringe components. When the common plunger is depressed, the fibrin sealer solution and the thrombin solution are combined in the nosecone, in equal volumes, to form the resulting fibrin sealant that is directly applied to the designated tissues.

The principle behind the use of fibrin glue is taken from wound healing wherein the first phase is inflammation which involves formation of thrombus through a series of events in the coagulation cascade. An indigenous preparation Reliseal (Reliance Industries, India) is also available with similar components. All fibrin sealants in use have two major ingredients, purified fibrinogen (a protein) and purified thrombin (an enzyme) derived from human or bovine (cattle) blood. Many sealants have two additional ingredients, human blood factor XIII and a substance called aprotinin, which is derived from cows’ lungs. Factor XIII is a compound that strengthens blood clots by promoting cross-linkage of fibrin strands. Aprotinin is a protein that inhibits the enzymes that break down blood clots.

## The technique for application

The two components of fibrin glue can either be applied simultaneously or sequentially, depending on the surgeons preference.

When simultaneous application is preferred, both the components are loaded into two syringes with tips forming a common port (Duploject syringe). When injected, the two components meet in equal volumes at the point of delivery. The thrombin converts the fibrinogen to fibrin by enzymatic action at a rate determined by the concentration of thrombin. The more concentrated thrombin solution, thrombin 500, produces a fibrin clot in about 10 seconds and the more dilute thrombin solution, thrombin 4, results in a clot in about 60 seconds after glue application to the surgical field. As mentioned earlier, both the extrinsic and the intrinsic mechanisms of blood coagulation are bypassed but the physiological final common pathway of coagulation is replicated. Factor XIII (present in the fibrinogen component of the glue) cross links and stabilizes the clot's fibrin monomers while aprotinin inhibits fibrinolytic enzymes, consequently resulting in a stable clot.

For sequential application, thrombin is first applied on to the area of interest, followed by a thin layer of fibrinogen. In a minute or two, coagulation starts and by two or three minutes, polymerization is complete.

Alternatively, when apposition is required between opposing surfaces, thrombin solution may be applied to one and fibrinogen to the other surface.

In all of these cases, prior to application of the glue, the surgical field must be dried meticulously. After application, the tissue is pressed gently over the glue for 3 minutes for firm adhesion. At the end of the procedure, pad and bandage is applied after instillation of antibiotic drops.

## Safety of Fibrin glue

Fibrin glue prepared from donor is as safe as other tested blood products.[27] Most but not all viruses can be inactivated by solvent / detergent treatment.[41,42]

The alternative approach to ensure that fibrin glue is virus free is by preparing it from homologous fresh frozen plasma from donors in whom current tests for viral markers are negative for at least six months after the donation.[47,48] This simple accreditation measure excludes the theoretical possibility of the donors having been in the “window period” when they donated blood or plasma. To further ensure its safety, most of the proteinaceous products are sterilized by gamma irradiation.

## Advantages

Fibrin glue reduces the total surgical time because time required to place sutures is saved.[6,27,52] The use of glue has been found to lower the risk of post-operative wound infection, contrary to conventional suturing.[52–54] This can be attributed to accumulation of mucous and debris in sutures which may act as a nidus for infection. However, there is no data available to substantiate the low incidence of post operative reaction and infection.

Mixtures of fibrin glue and antibiotics are being used for local delivery of antimicrobial activity.[53] It is well tolerated, non-toxic to the tissue wherever it is applied and has some antimicrobial activity. The smooth seal along the entire length of the wound edge results in a higher tensile strength, with the bond being resistant to greater shearing stress. Fibrin glue is also a useful adjunct to control bleeding in selected surgical patients.[54] It has a low incidence of allergic reactions. However, anaphylactic reactions following its application have been reported.[55,56] This reaction has been attributed to the presence of aprotinin in fibrin glue.

Fibrin glue encourages the formation of adhesions when applied to contaminated tissues. Its use in infected wounds[57,58] has been reported by two authors. This could be possible due to presence of aprotinin which possesses some antimicrobial activity.[59] Chen *et al*. however, reported that fibrin glue failed to demonstrate any bacteriostatic effects to either Gram-ve or Gram+ve bacteria by verifying the size of the bacterial growth inhibition.[60] They also detected minimal cytotoxic activity but this was not found to be significant clinically.

## Disadvantages

The major drawback to its use is the risk of transmitted disease from pooled and single-donor blood donors.[47,50] The same can be minimized to a great extent by obtaining the blood from screened healthy donors.[47,48] The safest preparation is by using the patient's own blood to prepare fibrin glue. It is expensive and autologous donation requires at least 24 hours for processing.The resultant product often has variable concentrations thereby resulting in an unpredictable performance. Moreover, tensile strength of fibrin glue has not been adequately determined and precludes quantification, being dependant on various extraneous factors also.

## Fibrin glue in ophthalmology

Both synthetic and biologic fibrin glues have a long history of use in ophthalmology.[5,6] Tisseel (Baxter Healthcare Corp, Deerfield II, USA) a commercially available fibrin biosealant has been used in Europe for more than 25 years in over 9.5 million surgical procedures.[61,62]

### Conjunctival surgery

As early as 1986 the glue was used in conjunctival surgery utilizing pericardium.[63] Currently, its use has gained popularity for both conjunctival closure and transplant.[64,65] A number of authors have tried its efficacy with favorable outcome.[66,67] Kheirkhah *et al*. performed amniotic membrane transplantation using fibrin glue in eyes with refractory conjunctivochalasis and achieved a complete/smooth/significant conjunctival surface in 44-56% of eyes.[68]

### In pterygium surgery

Ever since the introduction of fibrin glue in ophthalmology, its major use has been in pterygium surgery.[69–75] It is a safe and effective method for attaching conjunctival autografts for wound closure following pterygium surgery. Its use results in a shorter operating time, less post operative discomfort and inflammation.

Jiang *et al*. in 2008 evaluated the efficacy and safety of fibrin glue in conjunctival autograft fixation in primary pterygium compared with that of suturing.[73] They found that fibrin glue application takes significantly shorter operating time and associated with fewer post operative symptoms than a sutured graft, indicating the safety of the procedure. Studies have also demonstrated its efficacy for amniotic membrane graft fixation during pterygium surgery in terms of reduction of surgical time and post operative discomfort.[74,75] Srinivasan *et al*. reported that rubbing the eye can cause graft dehiscence following pterygium surgery with fibrin glue.[76]

### In strabismus surgery

Spierer *et al*. in 1997 carried out an experimental study using fibrin glue in strabismus surgery for conjunctival closure.[77]

An experimental study was carried out by Erbil *et al*. in 1991 where fibrin glue was used for conjunctival wound closure in place of classical sutures.[78] Histopathological study revealed better results with fibrin sealant than those with sutures. Biender *et al*. in 1996 conducted a clinical study on six patients who underwent squint surgery and concluded that conjunctival closure with fibrin glue was a good alternative to suturing the conjunctiva in strabismus surgery.[79] It also resulted in a more comfortable postoperative course.

Two comparative studies, suture versus fibrin glue, from India are reported, suggesting the efficacy of fibrin glue in conjunctival closure following strabismus surgery.[80,81] The recent study by Erbagei *et al*. emphasizes the role of fibrin sealant (Beriplast) as an attractive therapeutic modality in strabismus surgery.[82] Tonelli *et al*. in 2004 used it in a rabbit model to find the efficacy of the glue in Faden operation.[83] Though the outcome was good in their study, they commented that in small muscle recessions, the glue was not strong enough to overcome the contractive strength of the muscle.

### In Corneal surgery

#### a) Corneal perforation and melt

Lagoutte *et al*. in 1989 used the glue for perforated corneal ulcer and ulcer with impending perforation, descemetocele and extremely thinned out corneas.[84] Vrabec *et al.* commented that it can be used as an alternative to corneal/scleral tissue fixation in perforations smaller than 2mm.[85] According to Hick, fibrin glue and cyanoacrylate tissue adhesives are both effective in the closure of corneal perforations up to 3mm in diameter.[86] They used the glue in fixing the amniotic membrane in refractory and perforated corneal ulcers and found it to be a viable option. Fibrin glue provides faster healing and induces significantly less corneal vascularization. Further studies by Bernaur *et al*. in 1995, Duchesne *et al*. in 2001 and Sii *et al*. in 2005 highlighted its use in corneal melt.[87–89] Similarly, Sii *et al*. have emphasized its use in perforated hydrops.[90]

#### b) Amniotic membrane transplantation

Hick *et al*. in 2005 and Duchesne *et al*. in 2001 reported its use in amniotic membrane transplantation.[86,88] It was found to be safe and effective in fixing the amniotic membrane to the ocular surface.

Liu *et al*. used fibrin glue to fix a polymethyl methacrylate ring to an amniotic membrane patch on the ocular surface as a therapeutic contact lens.[91] They found it to be effective in alleviating the patient's pain and shortening surgical time.

Kheirkhah *et al*. performed amniotic membrane transplantation using fibrin glue in eyes with partial limbal cell deficiency and found it to be a safe and effective procedure.[92]

Sekiyama *et al*. evaluated the efficacy and safety of transplantation of fibrin glue coated freeze dried amniotic membrane (FD-AM) for ocular surface reconstruction.[93] They found out that the FD-AM retained most of its biological characteristics indicating that it was safe and efficacious for ocular surface reconstruction.

#### c) Lamellar keratoplasty

Rosenthal *et al*. used a platelet/fibrinogen/thrombin mixture to fixate the lamellar corneal graft in experimental animals.[94] It has also been used successfully in lamellar graft in highly vascularized and infiltrated corneas.[95–98]

#### d) Deep anterior lamellar keratoplasty

Narendran *et al*. carried out deep lamellar keratoplasty (DLK) using fibrin glue supported with overlay sutures.[99] They found it to be a time efficient and effective technique. They concluded that fibrin glue is ideally suited when both recipient bed and donor buttons are of same size and thickness.

#### e) Penetrating keratoplasty

Katzin in early forties introduced fibrin glue into ophthalmology and performed penetrating corneal grafts in rabbits.[6] This pioneering study was the inspiration behind various studies including those by Ignacio *et al*. in 2006 and Bahar *et al*. in 2007.[100,101] They reported that in “Top Hat” keratoplasty, fibrin glue was mechanically more stable than suturing. They used incisional bursting pressure of 185 mmHg (range 90-300 mm Hg) for testing stability, demonstrating that fibrin glue provides faster healing and induces significantly less corneal vascularization.

#### f) Limbal cell transplantation

Fibrin glue has also been used effectively and safely to fix the donor limbal lenticule on the bed of the recipient in cases of limbal deficiency. As suturing the thin lenticule in limbal transplantation creates special problems related to tissue apposition, suture related inflammation, vascularization and patient discomfort to the exposed sutures on the ocular surface, the use of glue was a rational alternative.[102]

#### g) Epikeratophakia

Using biological adhesive, Rostron *et al*. carried out an experimental comparative study with that of suturing the lenticules.[103] They calculated that the operating time reduced to 50% when glue was used instead of sutures. In another experimental comparitive study using both cyanoacrylate and fibrin glue, Brittain *et al*. found that the bond strength was 140 gm/cm.[104] Moreover, the bond strength of both, fibrin glue and cyanoacrylate glue was similar.

#### h) Temporary keratoprosthesis

Uhlig *et al.* used the fibrin glue as an aid to stabilize temporarily sutured keratoprosthesis.[105]

### Refractive surgery

#### a) Treating epithelial ingrowth

In the refractive arena, it is for epithelial ingrowth that the product has gained the most ground, typically in recalcitrant cases of epithelial ingrowth. The glue forms a mechanical barrier and prevents the epithelial cells from growing underneath the flap, at least until the flap is healed. The glue typically dissolves gradually over a two weeks period and by then, the epithelial surface and stromal interface show complete healing with no cells in the interface.

Anderson *et al*. in 2003 and Yeh *et al*. in 2006 suggested that additional use of fibrin glue in conjunction with debridement may be helpful in preventing recurrence of epithelial ingrowth.[106,107] Naravaez *et al*. in 2006 commented that even severe progressive epithelial ingrowth may be treated successfully with a combination of mechanical debridement, flap suturing and fibrin glue application.[108]

However, the major disadvantages of the use of fibrin glue for flap reattachment is that the glue is fairly opaque when it polymerizes and as a result it is difficult to see through it to determine if there are inflammatory cells in the interface. In addition, it is expensive and requires special equipment and preparation time.

While prevention of recurrent epithelial ingrowth is the most common refractive use for fibrin glue, it also has other possible applications. It is used like a bandage contact lens or as an ocular surface bandage. However, as the most rapid visual rehabilitation is required after LASEK or Epi-LASIK and glue does not form a good optical surface for vision, rather forms a rough Band-Aid, it is not advocated in routine LASEK or Epi-LASIK surgery.

#### b) As a temporary basement membrane

It is being used on photorefractive keratectomy operated corneas to reduce corneal haze.[109]

#### c) In flap tear /traumatic flap dislocation

Usually a flap tear occurs secondary to trauma, which induces some epithelial defect. In that scenario, it adheres better to the denuded surface on or around the flap and prevents epithelial ingrowth.[107]

### In glaucoma surgery

#### a) Conjunctival closure:

Its use as an effective method of achieving conjunctival wound closure in glaucoma surgery has been described by O'Sullivan *et al*. in 1996.[110]

#### b) Management of post operative leaking bleb:

Its successful use has been reported in the management of post trabeculectomy hypotony. A number of studies have been carried out both experimentally and clinically to prove its efficacy.[111–113]

#### c) In glaucoma drainage device (GDD) surgery:

It is considered as a safe substitute for some of the sutures used in GDD surgery. It does not alter intra ocular pressure (IOP) control, reduces postoperative conjunctival inflammation and reduces the surgical time. However, further studies are needed to better understand the role of the glue in GDD implantation. Vatimarki used fibrin glue intra-operatively in a series of 42 eyes subjected to GDD surgery.[114] Their results revealed that mean IOP on first post operative day was 30.5 ± 10 mm Hg which subsequently became within normal limits indicating that the intraoperative use of fibrin glue was a viable option for reducing peribulbar filtration and preventing immediate post operative hypotony after GDD surgery.

### Lens surgery

Its use in cataract surgery to close the capsular perforation[115] and cataract incision[116,117] has been tried since 1987. Bushmann in 1994 conducted both experimental and clinical studies to seal the traumatic perforation of anterior and posterior capsule by fibrin glue with successful results.[118] It has also been used for the prevention of post operative astigmatism and to seal the wound in small incision cataract surgery.[119,120] Recently, the glue is being used to fix the haptics of IOL to the tissue in place of sutures.[121]

### Vitreo retinal surgery

As early as 1988, Zauberman *et al.* have reported its use for conjunctival wound closure following retinal detachment surgery.[64] Mentens *et al*. in 2007 compared the efficacy of fibrin glue in comparison with conjunctival closure by sutures following 20 gauge needle pars plana vitrectomy in 504 eyes.[122,123] They commented that fibrin glue offers significantly better results than suturing for closure of conjunctival wounds. In another study, Batman *et al*. supported the view of Mentens and suggested that in case of persistance of leaking wound following transconjunctival sutureless vitreoretinal surgery, application of fibrin glue is a better alternative over suturing.[124] Its use has also been reported in macular hole surgery.[125]

## Lid and adnexal surgery

### a) Lid surgery

The glue was used in eyelid surgery for fixing the free autologous skin transplants for covering skin defects and the procedure is advantageous as early fibrovascular ingrowth into the transplant is stimulated. It is also helpful in lid split procedure combined with free skin graft for severe upper eyelid entropion. In lower eyelid trichiasis, glue has been used for fixation of free autologous conjunctival transplants from the upper fornix after separation of the lashes from the posterior lamella with a lid split technique.[126,127]

### b) Lacrimal surgery

It has been used for reconstructing lacerated canaliculi,[128] in canaliculocystotomy, canaliculodacryocystorhinostomy, for the microanastomosis between canaliculi and lacrimal sac and for attaching lacrimal and nasal mucosal flaps.[129]

### Plastic, reconstructive and orbital surgery

Gosalin *et al*. used fibrin glue to attach soft tissue in oculoplastic surgery.[130] In orbital surgery it has been used to fix the secondary orbital implant. It has also been used in plastic and reconstructive surgery.[130,131]

### Drug delivery

The suitability of existing topical fibrin glue preparations for local drug delivery is greatly limited because of the limited capacity of fibrinogen to actively bind growth factors or other therapeutic agents.

## Conclusions

As the use of sutures is fraught with complications, bioadhesives have emerged as a viable alternative for tissue cooptation, over the last three decades. In summary, the advantages of fibrin glue that makes it so useful in ophthalmolgy are:

- It reduces surgical time
- Adequate bond strength, good sealent, safe, minimal allergic or toxic reactions and minimizes bleeding
- Easy to undo
- Disappears eventually
- Can plug perforations
- Excess amount can be trimmed
- Does not produce inflammation

However, further studies are required before fibrin glue is inducted in standard ophthalmological practice in the place of sutures.
